# An unusual case of linear IgA disease affecting only the oral gingiva: a case report

**DOI:** 10.1186/s12903-023-03250-1

**Published:** 2023-08-05

**Authors:** Jianing He, Jun Shen, Wei Guo

**Affiliations:** 1 Department of VIP Service Center, Stomatological Hospital, Southern Medical University, 366# Southern Jiangnan Road, Guangzhou, 510280 Guangdong China; 2 Department of Oral Pathology, Stomatological Hospital, Southern Medical University, Guangzhou, 510280 Guangdong China

**Keywords:** Linear IgA disease, desquamative gingivitis, Blister, Case report

## Abstract

**Background:**

We present a case report on desquamative gingivitis diagnosed as linear IgA disease (LAD), which is a rare autoimmune bullous disease exclusively affecting the oral gingiva. The oral mucosa can be impacted by various autoimmune bullous diseases, and our report focuses on this particular manifestation of LAD.

**Case presentation:**

This patient presented with atypical symptoms, as frequent blister formation on the gingiva was the primary clinical manifestation. A combination of systemic and local treatment was administered to the patient. Following the treatment, there was a significant improvement observed in both the erythema and the bullous lesions on the gingiva.

**Conclusions:**

A more suitable local treatment strategy should be formulated for patients presenting with oral topical lesions, which clinicians can employ effectively.

## Background

Desquamative gingivitis (DG) is a descriptive term used to indicate epithelial desquamation, erythema, erosion, and/or vesiculobullous lesions of the attached and marginal gingiva [1]. DG may be a manifestation of several mucocutaneous diseases, most commonly mucous membrane pemphigoid (MMP), pemphigus vulgaris (PV) and lichen planus [2–4].

Linear IgA disease (LAD) is a rare autoimmune blistering disease characterized by antibodies of linear IgA deposits along the basement membrane zone [5]. LAD occurring exclusively in the oral mucosa is relatively uncommon. This report presents a rare case of linear IgA disease that solely manifested as desquamative gingivitis. The primary focus of this report lies in discussing the local treatment options.

## Case presentation

A 54-year-old Chinese woman was referred to our clinic by her general dentist. The patient had been experiencing gingival blisters and soreness for over 2 years. Previously, her general dentist had administered a single intralesional steroid injection into the gingiva, which provided temporary relief but the symptoms recurred soon after. The patient had a childhood diagnosis of favism (glucose-6-phosphate dehydrogenase deficiency, G-6-PD deficiency). Apart from this condition, the patient was in good health and had no known allergies. She denied experiencing any cutaneous, genital, or ocular symptoms, and she had not taken any prescribed or illicit drugs prior to the onset of the oral lesions.

**Fig. 1:**
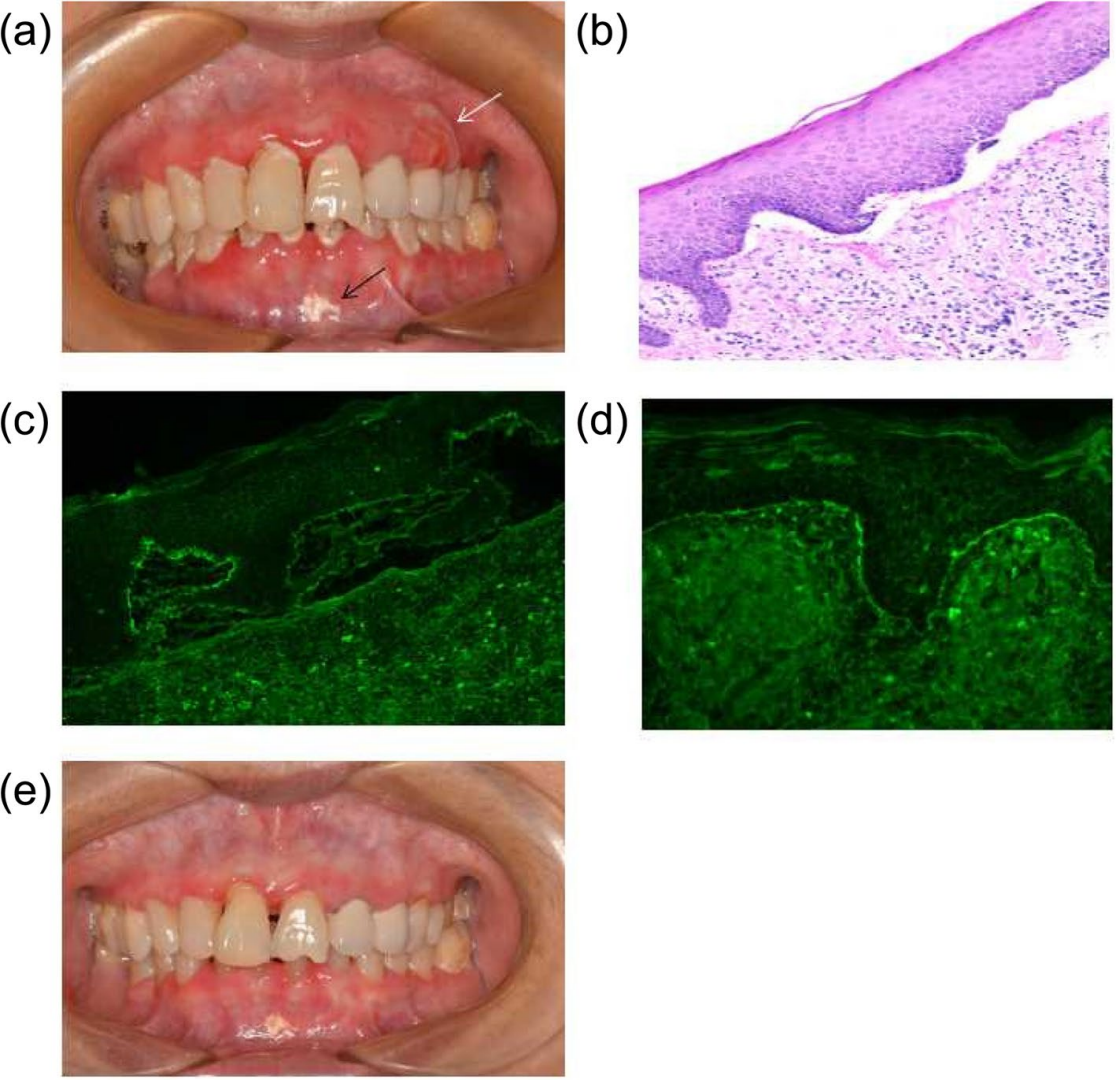
Erythema and vesiculobullous lesions were observed on the buccal side of the gingiva (indicated by the white arrow), while a white spot was present in front of the labial gingiva of the mandible (indicated by the black arrow) (**a**). Histological examination of gingival tissue with a bullous lesion revealed a subepithelial split with mixed inflammatory infiltration (hematoxylin and eosin, × 40) (**b**). DIF examination demonstrated a linear deposition of IgA (**c**) and C3 (**d**) along the basement membrane. An image of the gingiva at the 1-year follow-up is shown (**e**)

Upon examination, the patient exhibited widespread erythema along the gingival margins. On the buccal aspect of the maxillary gingiva, there was easy peeling of the epithelium, accompanied by the presence of vesiculobullous lesions (Fig. 1a). Nikolsky's sign was positive. A white spot was observed in front of the labial gingiva of the mandible (Fig. 1a). Furthermore, there was evidence of plaque-associated periodontitis affecting all of her teeth.

A gingival biopsy was conducted and sent for routine histological examination as well as direct immunofluorescence (DIF) studies. The histological examination revealed a subepithelial split accompanied by a mixed inflammatory infiltration (Fig. 1b). The DIF analysis demonstrated a linear deposition of IgA (Fig. 1c) and C3 (Fig. 1d) along the basement membrane. Based on the clinical presentation, histological findings, and DIF results, a diagnosis of LAD was established.

The patient received systemic prednisone treatment at a daily dosage of 30 mg (equivalent to 0.5 mg/kg) and visited the clinic every 2 weeks. Additionally, intralesional injections of triamcinolone were administered twice in the vestibular groove of the gingiva to alleviate pain while eating. After a month, significant improvement was observed in the erythema and bullous lesions on the gingiva, allowing for a gradual tapering of the prednisone dosage. The patient maintained oral hygiene and received antifungal prophylaxis through alternate use of daily mouth rinses containing chlorhexidine and sodium bicarbonate. Treatment for her periodontitis was initiated after the resolution of the gingival lesions. At the one-year follow-up, the patient's condition remained stable, with a maintenance dose of prednisone set at 2.5 mg daily (Fig. 1e). No significant adverse event was observed during the course of systemic corticosteroid treatment.

## Discussion

LAD primarily affecting the gingiva is a rare occurrence. The clinical manifestation of oral LAD is nonspecific, with previously reported cases predominantly involving characterized by gingival congestion and ulceration [6]. The noteworthy aspect of the present case is the frequent occurrence of blisters on the gingiva. The gold standard for diagnosis of oral LAD is DIF [7], with the characteristic feature of continuous, linear deposition of IgA along the basement membrane [5]. The DIF analysis in the present case revealed a linear deposition of both IgA and C3 along the basement membrane, consistent with the diagnosis of LAD. The main differential diagnoses for oral LAD include mucous membrane pemphigoid (MMP) and pemphigus vulgaris (PV), both of which can also present as vesiculobullous lesions in the gingiva. However, it is important to note that in MMP and PV, the major type of autoantibody is IgG [8, 9]. Therefore, distinguishing LAD from these diseases can be achieved by comparing the fluorescence intensity of IgA and IgG against the immunopathological features. However, there is ongoing debate regarding the interpretation of DIF findings in LAD. Some researchers define LAD strictly as the linear deposition of IgA in the absence of other immunoreactants [10]. When both IgA and other immunoreactants are present, differentiating LAD from other subepidermal blistering diseases becomes challenging. In our case, we diagnosed it as LAD since IgG, which is the predominant antibody in most pemphigoid cases, was not detected. However, considering the potential clinical, histological, and immunological overlaps among different subepidermal blistering disorders, there is a need for a more standardized and stringent diagnostic criteria to be established for clinicians.

Skin lesions in LAD can often be managed with potent topical steroids [11]. However, in the oral cavity, the efficacy of topical drugs can be compromised due to poor adherence to affected sites, resulting in a shorter duration of action. Intralesional steroid injections have been considered as an alternative approach. However, in this particular patient, intralesional injections had negative consequences. A white spot, resulting from irregular injections, was observed in front of the labial attachment gingival of the mandible in Fig. 1a. The drug was deposited in the attachment gingival and could not be metabolized effectively. It should be noted that the attachment gingival, lacking a submucosal layer and having fewer blood vessels in the lamina propria, is not a suitable site for intralesional injections. We recommend administering labial gingival injections in the area of the corresponding vestibular groove rather than the attached gingiva due to its rich blood circulation. Systemic therapy was also necessary for this patient due to the frequent occurrence of vesiculobullous lesions. The majority of LAD patients show a positive response to systemic dapsone, prednisone, or sulfonamides [11]. In our case, the patient was unable to take dapsone due to G-6-PD deficiency. Effective control was achieved with a low-dose of prednisone (30 mg daily) alone.

Nonsurgical periodontal therapy, including dental scaling and detailed oral hygiene instructions improved both periodontal clinical parameters and the severity of DG lesions or symptoms. Numerous reports highlight the significance of early periodontal intervention in cases of desquamative gingivitis [12]. However, we respectfully disagree with this perspective because, in the majority of cases, the affected gingival epithelium is extremely fragile and prone to detachment even with minor trauma, including gentle brushing. Currently, there is no direct evidence indicating a causal relationship between periodontitis and desquamative gingivitis. Therefore, we propose that any local periodontal treatment should only be conducted once the gingival blistering or peeling has been effectively controlled. In our patient's case, a thorough professional cleaning was performed after one month of prednisone therapy when the gingival condition had significantly improved, reducing the risk of gingival injury during periodontal treatment.

## Conclusions

In summary, we presented a rare case of linear IgA disease that solely manifested as desquamative gingivitis. The diagnosis was confirmed through histopathological and immunopathological examinations. A comprehensive treatment regimen comprising systemic and local intervention was implemented for the patient. It is crucial to develop more integrated treatment guidelines specifically tailored for these cases where lesions are localized to the oral cavity.

## Data Availability

The datasets used and/or analysed during the current study available from the corresponding author on reasonable request.

## Abbreviations

LAD: Linear IgA disease
G-6-PD deficiency: Glucose-6-phosphate dehydrogenase deficiency
DIF: Direct immunofluorescence
